# Is Emotion recognition processing across menstrual cycle and a history of Postpartum Depression potential risk factors for Premenstrual Dysphonic Disorder?

**DOI:** 10.1192/j.eurpsy.2022.2223

**Published:** 2022-09-01

**Authors:** A. Vardiampasis, C. Gramandani

**Affiliations:** 1 GENERAL HOSPITAL OF RETHYMNO, Mental Health Center Of Rethymno, RETHYMNO, Greece; 2 GENERAL HOSPITAL OF RETHYMNO, Mental Health Center, ΡΕΘΥΜΝΟ, Greece

**Keywords:** PMDD, PPD, ERT

## Abstract

**Introduction:**

Many women during the different phases of the menstrual cycle experience significant emotional and cognitive changes; for some, these changes can affect their everyday living. Premenstrual Dysphonic Disorder (PMDD) is a health problem that affects up to 5% of women of childbearing age. The exact cause is unknown; still, hormonal changes throughout the menstrual cycle may play a role. Women with a family history of Postpartum Depression (PPD) may be at increased risk.

**Objectives:**

The purpose was to examine if Emotion recognition processing across menstrual cycle and a history of PPD are potential risk factors for PMDD.

**Methods:**

We identified 34 women with a history of PPD and contacted their daughters to explain the purpose of our study. Of those meeting the criteria to participate, 38 volunteered (aged 18-30 y.o., right handed, educational level >9 y., regular cycle duration). The Emotion Recognition Task (ERT) was administered in the luteal and the follicular phase. Women found to present significant differences in emotion recognition depending on the estradiol and progesterone levels were clinically interviewed (DSM-V).

**Results:**

Of the 16 women who have showed significant differences across the two phases of the menstrual cycle, 7 were diagnosed with PMDD (43,7%). Among the ones who have not presented differences (22), only 2 received a diagnosis of PMDD (9%).

**Conclusions:**

This study shows that Emotion recognition processing across menstrual cycle and a history of PPD may predict which women could be at risk for PMDD, playing, therefore a key role in PMDD early diagnosis.

**Disclosure:**

No significant relationships.

